# Simultaneous Assessment of Speech Identification and Spatial Discrimination: A Potential Testing Approach for Bilateral Cochlear Implant Users?

**DOI:** 10.1177/2331216515619573

**Published:** 2015-12-30

**Authors:** Jennifer K. Bizley, Naomi Elliott, Katherine C. Wood, Deborah A. Vickers

**Affiliations:** 1 University College London, London, UK

**Keywords:** speech, localization, normal hearing, hearing impaired, cochlear implant

## Abstract

With increasing numbers of children and adults receiving bilateral cochlear implants, there is an urgent need for assessment tools that enable testing of binaural hearing abilities. Current test batteries are either limited in scope or are of an impractical duration for routine testing. Here, we report a behavioral test that enables combined testing of speech identification and spatial discrimination in noise. In this task, multitalker babble was presented from all speakers, and pairs of speech tokens were sequentially presented from two adjacent speakers. Listeners were required to identify both words from a closed set of four possibilities and to determine whether the second token was presented to the left or right of the first. In Experiment 1, normal-hearing adult listeners were tested at 15° intervals throughout the frontal hemifield. Listeners showed highest spatial discrimination performance in and around the frontal midline, with a decline at more eccentric locations. In contrast, speech identification abilities were least accurate near the midline and showed an improvement in performance at more lateral locations. In Experiment 2, normal-hearing listeners were assessed using a restricted range of speaker locations designed to match those found in clinical testing environments. Here, speakers were separated by 15° around the midline and 30° at more lateral locations. This resulted in a similar pattern of behavioral results as in Experiment 1. We conclude, this test offers the potential to assess both spatial discrimination and the ability to use spatial information for unmasking in clinical populations.

## Introduction

Bilateral cochlear implantation is routine standard of care for many health-care systems across the world (Peters, Litovsky, Lake, & Parkinson, 2004). In 2009, in the United Kingdom, the National Institute for Health and Care Excellence (NICE) developed guidelines (NICE, 2009) suggesting that all hearing-impaired children and adults with both a hearing and visual impairment should receive bilateral cochlear implants (CIs). With the rise in the provision of bilateral CIs, there is a need to be able to assess the binaural perception of individuals with two devices in a fast and straightforward way. However, children are often cognitively and linguistically too young to undergo many of the assessments of speech in noise and spatial perception and, even when assessments are age-appropriate, the test battery is too time consuming to be clinically viable.

To conduct traditional spatial release from masking (SRM) tasks, three test conditions are typically conducted to assess performance of both ears. Effective measurements of SRM require that speech identification is assessed (a) with both speech and noise from a frontal speaker, (b) with the speech from in front but the noise spatially separated from a speaker at 90° on the left, and finally (c) with the speech from in front and the noise spatially separated from the speech at 90° from the right speaker (Litovsky, 2005). An alternative configuration can be used with the spatially separated noise being presented from both speakers that are 90° on either side of the listener. This has the advantage of being quicker but does not provide information on the effectiveness of each individual ear. Traditional measurements of SRM provide a great deal of information. Depending on the spatial configurations tested and the analyses used, such measures can provide information about both the binaural (summation and squelch) and monaural (head shadow) contributions to speech intelligibility in spatially separated noise.

Localization tasks typically involve pointing to the loudspeaker that appears to present a sound. This measure provides a reliable estimate of the accuracy and precision with which a listener can locate a sound in space. However, when testing children, the reliability of standard sound localization tasks can be reduced particularly when testing smaller speaker separations (15°). To overcome this reduction in reliability, a greater number of trials are required to ensure that findings are valid (Lovett, Kitterick, Huang, & Summerfield, 2012); this inevitably results in an increase in test time, which can be problematic when testing young children with shorter attention spans. There has been some success demonstrating benefit in lateralization tasks that many children find less taxing: Vincent et al. (2012) used a lateralization paradigm that used speakers at ±30° as well as more standard ±90° approaches, which enabled assessment of finer spatial measures. Lovett et al. (2012) looked at the age-appropriateness of different spatial measures and reported that children as young as 1.5 years could perform movement tracking tasks and from 3 years could perform lateralization tasks. There is evidence that from 5 years and upward standard localization tasks can be completed (Van Deun et al., 2009).

Combined assessment of SRM and sound localization involves two time-consuming tests and consequently results in an impractically long testing period. Other patient groups including older adults or those with cognitive or linguistic difficulties could also struggle to complete a large and taxing test battery. These individuals could benefit from the simplification necessary for evaluating young children, and if such a test was sufficiently quick and effective, it would enable routine clinical evaluation of binaural hearing to take place.

While some tests of spatial discrimination have used speech as a stimulus, they have rarely also tested speech perception. The choice of stimulus used in localization/lateralization tasks is important, as both the spectral content and the relevance of the stimulus to the listener may influence task performance. Noises and tone bursts are not of interest to a young child, and it is more appropriate to use a more meaningful or engaging signal when testing young children. Vincent et al. (2012) used environmental sounds, and other researchers have used speech as a stimulus (Begault & Wenzel, 1993; Begault, Wenzel, & Anderson, 2001; Jones, Kan, & Litovsky, 2014; Kobler & Rosenhall, 2002; Lovett et al., 2012; Ricard & Meirs, 1994). Ricard and Meirs (1994) tested both spatial unmasking and sound localization using speech stimuli presented in virtual acoustic space. Kobler and Rosenhall (2002) developed a full circle localization task in which speech perception scores were also determined together with localization abilities for a group of adult hearing aid users with mild-to-moderate impairment. This approach was effective and enabled both abilities to be assessed in one task for this group of adults. Such studies have not been conducted with young children, and it seems likely that for children or those with poorer perceptual abilities, such a task could be too complex and demanding. However, components of this approach could assist with the development of a task that could be used with young children.

Typically, measures of speech intelligibility in noise using SRM paradigms do not exhibit a relationship between speech perception and localization ability (Rychtáriková, Van den Bogaert, Vermeird, & Wouters, 2011), suggesting that the two assessments are testing different aspects of spatial hearing. Both assessments suffer from some limitations—as discussed earlier, children in particular struggle with sound localization tasks. The SRM measure is a good way to look at the impact of moving the noise source away from the target speech but always for a target from the front. SRM can be used to provide some information on binaural summation and certain unmasking conditions, as well as monaural (head shadow) contributions to speech intelligibility in spatially separated noise. However, SRM assesses abilities in a static situation. For many perceptual experiences it is useful to understand speech arriving from different directions and being aware of moving sources can also be important from a safety perspective. To fully understand binaural processing capabilities, it is essential that both SRM and spatial discrimination measures are assessed to provide a more refined measure of spatial hearing than that derived from using an SRM approach alone. The current tests of localization ability that are feasible to be conducted clinically do not appear to deliver this for children.

In the current climate of delivering bilateral CIs as a routine intervention with a great deal of clinical concern (as highlighted by this special issue) about the approaches used for fitting, there is a need to develop an assessment measure that provides meaningful, ecologically valid information on binaural processing ability that are sensitive to small modifications in fitting. An assessment that not only uses speech tokens as a stimulus but also assesses spatial speech perception at the same time as localization, without the need for a large SRM test battery, would be ideal. To this end, this project aims to develop an assessment approach that can ultimately be used with a wide range of participant groups from young children to elderly listeners to evaluate binaural hearing ability. Our objectives were to develop a single test that could simultaneously measure speech discrimination from multiple locations, spatial unmasking, and spatial discrimination. We aimed to develop a test that was ecologically valid by using speech as stimuli and a multisource background noise. We required that this test should be sensitive enough to be able to demonstrate expected differences in spatial listening abilities throughout space and to measure changes in performance across time or across different intervention strategies. To achieve this aim, we adapted a recently developed relative spatial discrimination task that required listeners to judge the relative location of two sequentially presented sound sources (Wood & Bizley, 2015) to enable combined testing of spatial discrimination and speech discrimination in noise.

## Methods

### Participants

This experiment received ethical approval from the UCL Research Ethics Committee (3865/001). Ten normal-hearing adults between the ages of 20 and 25 participated. All participants had normal-hearing thresholds as assessed by pure tone audiometry and had no reported neurological disorders.

### Testing Chamber

**Figure 1.**
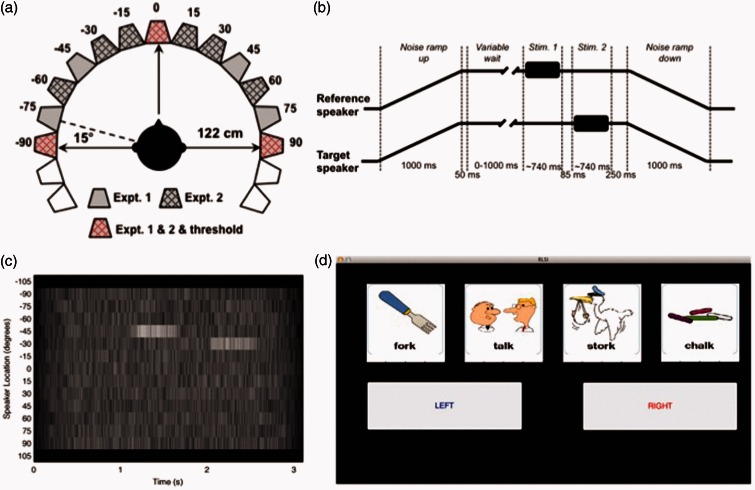
Methods. (a) Testing chamber. The location of the speakers used in Experiments 1 and 2 are marked. (b) Task timeline showing the main elements of a single trial: First, independent babble ramps up over a 1-s period, across all speakers. After a variable wait two speech tokens are presented, one from each of two adjacent speakers separated by a silent interval of >85 ms. Participants are allowed to respond any time after the end of the second word sound. The babble then ramps down to silence over a 1-s window. The next trial begins automatically after the subject has made their response. (c) An image showing the typical voltage output across all speakers for a single trial in which speech tokens (reference and target) are presented from the speakers at −45° and −30°. White indicates a more intense sound. The salient features are the independently generated babble from each speaker, the common onset and offset ramps applied to all speakers and the reference and target words (here at a level equivalent to +11 dB SNR). (d) The GUI that participants saw on a touch screen. Participants performed a simultaneous closed-set word identification task and relative localization task. All four possible words from each group (listed in Table 1) were displayed on the GUI with pictures. Participants made their response by touching the two words that they had heard, in the correct order, followed (or preceded by) pressing either left or right to indicate that the target word had originated from the left or right of the preceding reference. GUI = graphical user interface; SNR = signal-to-noise ratio.

For testing, participants sat in the middle of an anechoic chamber (Figure 1(a), 3.6 × 3.6 × 3.3 m: width × depth × height) with sound-attenuating foam triangles on all surfaces (with dimensions of 24 cm triangular depth and 35 cm total depth) and with a suspended floor. The participant sat in the center surrounded by a ring of 18 speakers, which were 122 cm from the center of the participant’s head and at ear level arranged at 15° intervals from −127.5° to +127.5° (Figure 1(a)). Participants sat on a chair with their head at the center of the chamber and with a touch screen tablet for recording responses on their laps.

### Stimuli

All stimuli were generated and presented at a sampling frequency of 48 kHz. Stimuli were monosyllabic word tokens from the Chear Auditory Perception Test (CAPT; Marriage, Vickers, Baer, & Moore, 2011) spoken by a single female British English talker. The CAPT was selected because the vocabulary was appropriate for young children. Sixteen tokens were used, divided into four groups (see Table 1) each of which targeted a particular type of discrimination to include complex vowel discrimination, simple vowel discrimination, initial contrastive consonant, and the final contrastive consonant. The utterances were between 445 and 885 ms (mean ± *SD* = 660 ± 102 ms) long with a variable amount of silence at the end (78 ± 97 ms).

**Table 1.** Word groups used in the speech identification task.

Discrimination	Abbreviation				
Complex vowel	Vc	Pale	Pool	Pile	Peel
Simple vowel	Vs	Hoot	Heat	Heart	Hurt
Initial consonant	Ci	Chalk	Talk	Fork	Stork
Final consonant	Cf	Cheat	Cheese	Cheap	Cheek

The design of the experimental paradigm was adapted from Wood and Bizley (2015). The first speech token (denoted the *reference*) was presented from one speaker. After a silent interval of at least 45 ms (mean 163 ± 97 ms), a second speech token was presented from an adjacent speaker (the *target*, Figure 1(b)). Participants were required to indicate, using the touch screen, the two words in the correct order and whether the target word was presented to the left or right of the reference word. Words were presented in a background of multitalker male babble generated by overlaying 4-word passages from 16 individual talkers drawn from Mark Huckvale’s SCRIBE database (www.phon.ucl.ac.uk/resource/scribe/). For each speaker and each trial, a random sample of the resulting babble was presented such that uncorrelated babble occurred from 13 independent locations (±90°, in 15° intervals) around the listener (Figure 1(c)). The overall level of noise was simultaneously ramped on and off with a linear ramp over 1 s, for all 13 noise sources, according to the schematic in Figure 1(b). The reference and target words could occur any time between 50 ms and 1,050 ms after the babble levels reached their maximum (i.e., 1050–2050 ms after trial onset). In these experiments, each speaker generated babble at 41 dB sound pressure level (SPL) and the mean noise level when all speakers were presenting the background noise was 52 dB SPL (calibrated using a CEL-450 sound level meter). The words were presented such that the vowels were matched in sound level resulting in a ±2.3 dB variation in absolute presentation level. Stimuli were presented by Canton Plus XS.2 speakers (Computers Unlimited, London) via a MOTU 24 I/O analogue device (MOTU, MA, USA) and 2 Knoll MA1250 amplifiers (Knoll Systems, WA, USA). The individual speakers were matched for level using a CEL-450 sound level meter, and the spectral outputs were checked using a Brüel and Kjær 4191 condenser microphone placed at the center of the chamber where the subject’s head would be during the presentation of a stimulus. The microphone signal was passed to a Tucker Davis Technologies System 3 RP2.1 signal processor via a Brüel and Kjær 3110-003 measuring amplifier. All speakers were matched in their spectral output that was flat from 400 to 800 Hz, with a smooth, uncorrected 1.2 dB/octave drop off from 400 to 10 Hz, and a smooth uncorrected drop off of 1.8 dB/octave from 800 Hz to 25 kHz. The MOTU device was controlled by MATLAB (MathWorks) using the Psychophysics Toolbox extension (Brainard, 1997; Kleiner et al., 2007).

### Threshold Estimation

Our goal in this study was to develop a listening task that would enable combined testing of speech discrimination in noise and spatial listening abilities and that would be both efficient and sensitive, to assess hearing in complex listening situations. Our long-term goal is to develop this test for clinical situations such as assessing the outcome of interventions such as binaural cochlear implantation or for comparing different signal processing methods in either CIs or hearing aids. We therefore included for each participant a short *threshold* test that allowed them to familiarize themselves with the procedural aspects of the task and enabled the normalization of difficulty across listeners. By matching the difficulty in this manner, we hoped that we would ensure that listeners were able to perform the main experiments at a level that avoided floor and ceiling effects. As well as serving to equalize difficulty across participants, this test additionally provided participants with procedural training in the task. As in the main experiments, participants heard two sequentially presented speech tokens and had to report, from a closed set of four options, which two words they heard in the correct order, as well as whether the second word originated from the left or right of the first word. In the threshold task, the first word always originated from the speaker at 0° azimuth, while the second word could occur at either 90° left or right of the midline. Participants performed this task at 6 signal-to-noise ratio (SNR) values spanning a 12.5 dB SPL range.

We reasoned that because a 90° shift (or 180° discrimination) in location far exceeds the minimum audible angle, a correct localization judgment serves to indicate that participants were able to detect the sound above the noise level. In Wood and Bizley, the 95% correct discrimination level was set as threshold, as we wanted to ensure that all of our stimuli were audible. The pilot testing for this experiment demonstrated that estimating the 50% correct speech discrimination threshold at 0° provided a more sensitive estimate of participants’ performance. When averaged across participants, this resulted in a mean localization performance of 95% correct, but individuals were rather variable—importantly, for a small number of individuals, localization performance was at ceiling at all SNRs tested, including at SNRs where speech discrimination thresholds were close to (or) at chance levels. Our aim was to ensure that people were operating at the same point on the psychometric function. We selected the 50% point on the speech identification psychometric because we wanted the majority of scores to fall above chance plus the critical difference for the CAPT (18% (Vickers et al., 2013)) and below 100% minus the critical difference, that is, 43% to 82%.

**Figure 2.**
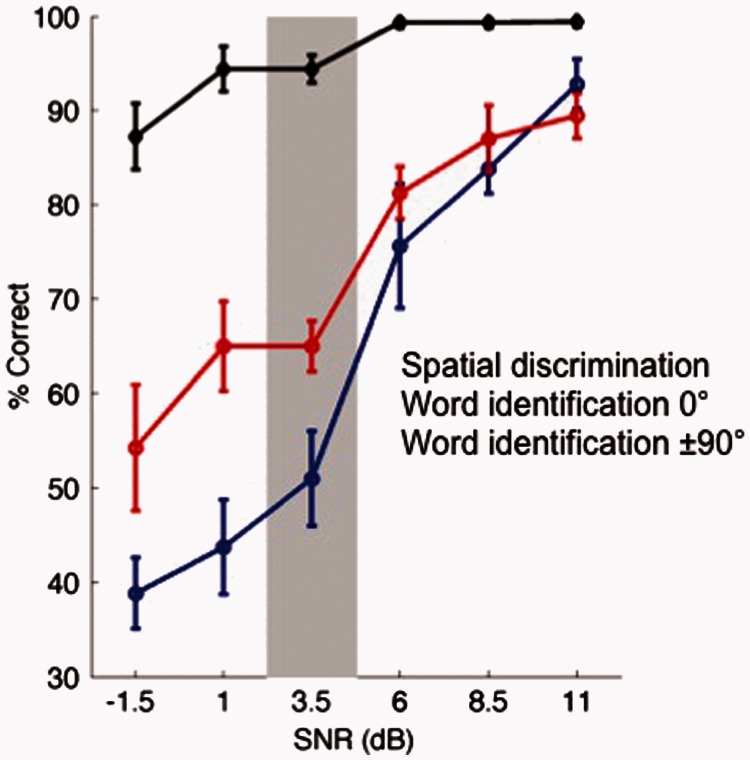
Threshold data. Threshold data for all participants (*n* = 10). In the threshold task, all reference words originated from the speaker at 0° and all target words from ± 90°. Participants therefore made a 180° spatial discrimination (black line, mean % correct ± *SEM*). Performance in the relative localization judgment (black) as well as word recognition (red, ±90° and blue, 0°) declines with less favourable SNRs; however, speech identification performance is worse for words presented at 0° than for words presented at 90°. For subsequent testing, each subjects’ threshold was individually determined based on their 50% correct point for word identification at the 0° speaker. The across subject testing level (mean ± *SE*) is indicated in gray. SNR = signal-to-noise ratio.

During the threshold test, participants heard two repetitions of each word for each SNR and performed eight trials in each direction and SNR combination, presented pseudorandomly, over a single testing block. SNRs ranged from +11 dB to −1.5 dB in 2.5 dB steps. Percentage correct word identification scores at the central (0°) speaker were fit with a binomial logistic regression function and the 50% detection threshold was extracted from the fitted psychometric function. The resulting threshold value determined the SNRs at which the main experiment was conducted. Across participants, this was 3.5 dB ± 1.23 (mean SNR ± standard deviation), as shown in Figure 2. The threshold task took roughly 20 min for subjects to complete.

### Testing

During testing, on each trial, the reference sound was presented from one of the speakers in the ring (where the speaker was selected pseudorandomly from the set of speakers used in that experiment, see Figure 1(a) and experimental methods in the following for speakers used in Experiments 1 and 2), and the target was presented from an adjacent speaker, either to the left or right (always a 15° change in location for Experiment 1, and either a 15° or a 30° shift in location for Experiment 2). The touch pad displayed a graphical user interface (GUI) which displayed, on the top row, four pictures, with the four possible words from that word group written underneath (Figure 1(d)) and on the bottom row were two buttons labeled left and right. Participants were instructed to select the two words that they heard in the correct order and to report the direction of the location shift between the first word and the second word. Participants were informed that they could identify the words or the direction in either order (although all reported after testing that they found reporting the words and then direction to be the most intuitive). Each trial began automatically 1 s after the participant made a response in the preceding trial. Subjects were instructed to perform the task as accurately as possible and had unlimited response times. Testing runs were divided into blocks lasting approximately 4 to 5 min. At the end of each block, the participant took a break before the next block. Testing took approximately 35 min in total for Experiment 1 and roughly 25 min for Experiment 2.

### Experiment 1: Simultaneous Assessment of Speech Identification and Sound Localization

In this task, all speakers in the frontal hemifield were used such that the testing locations were ±90°, ±75°, ±60°, ±45°, ±30°, ±15°, and 0°. Participants performed two repetitions of every word from each pair of locations, yielding 16 trials for each direction judgment and a single presentation of every word at every speaker location.

### Experiment 2: Simultaneous Assessment of Speech Identification and Sound Localization Using a Restricted Set of Speaker Locations

To determine whether the test outlined in Experiment 1 would be sensitive in a clinical situation, we repeated Experiment 1 using a reduced subset of speakers equating to those used in the Crescent of Sound (Kitterick, Lovett, Goman, & Summerfield, 2011). These were ±90°, ±60°, ±30°, ±15°, and 0° (see Figure 1). Otherwise all experimental procedures were identical. The order in which participants underwent Experiments 1 and 2 was counterbalanced.

### Analysis

Performance was assessed by calculating the percentage of correct responses for each judgement. In determining word recognition accuracy, we considered the first and second stimulus intervals to be equivalent: Accuracy of word identification did not differ for words presented in the first (reference) or second (target) intervals (mean ± *SD*; accuracy for words in the first interval, 63.4% ± 10.4%; accuracy in the second interval, 71.0% ± 6.3%; paired *t* test, *p* = .61). Statistical analyses were performed in SPSS (IBM).

## Results

Listeners first completed the threshold test, before completing the main experiments.

**Figure 3.**
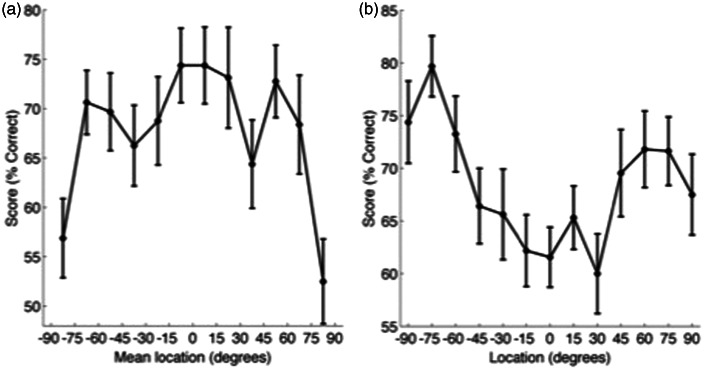
Effect of azimuth on task performance. (a) Relative localization performance (% correct, mean ± *SE*) by mean reference-target speaker location. (b) Word identification performance by speaker location.

First, the effect of the azimuthal location on listeners’ ability to perform the spatial discrimination task and to identify speech in noise was determined. In Experiment 1, all speaker pairs were separated by 15°. Normal-hearing adult listeners tested in a previous spatial discrimination task with noise-burst stimuli showed a characteristic pattern of performance with the highest spatial acuity around the midline and a decrease in performance at more lateral locations (Wood & Bizley, 2015). For broadband stimuli, such as speech, this decline in performance is marked only at the most peripheral locations. Figure 3(a) plots the spatial discrimination abilities of listeners in Experiment 1. Performance is plotted relative to the mean location of each pair of speakers (e.g., if the first sound was presented from the 15° to the right and the second at 30° to the right (or vice versa), the score is plotted at 22.5° to the right). This means that for each point in space, listeners judged an equivalent shift in location with an identical magnitude of change in binaural localization cues. In keeping with previous investigations with spectrally rich sounds, spatial discrimination ability assessed using speech stimuli (Figure 3(a)) also shows a modest variation through space with the best performance evident around the midline and a decline in performance at the most peripheral speakers tested (repeated measures analysis of variance [ANOVA], *F*(11,99) = 3.66, *p* < .001; post hoc tests reveal the peripheral (±82.5) locations to be significantly lower performance than the central locations). In contrast to spatial discrimination ability, normal-hearing listeners show an inverse pattern of word recognition ability (Figure 3(b)): Performance peaks at ±75° and is lowest at the midline (repeated measures ANOVA, *F*(12,108) = 5.40, *p* < .001; post hoc tests showed significant differences between peripheral and central locations).

**Figure 4.**
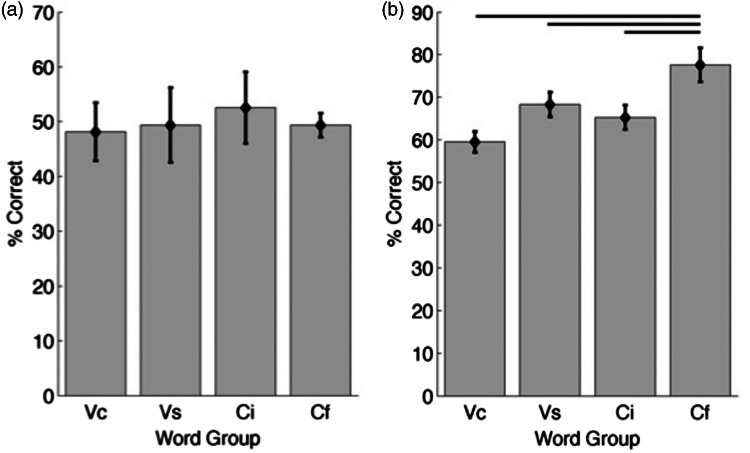
Effect of word group on task performance. (a) Word identification performance by word group (% correct, mean ± *SE*). Bars indicate significant pairwise post hoc differences. Chance performance is 25%. (b) Relative localization performance by word group. Chance performance is 50%.

Listeners discriminated four groups of speech sounds, each of which required that participants were able to make a particular type of discrimination: Two groups targeted vowel sounds (both complex and simple), and two groups targeted consonant sounds either at the beginning or the end of the word. These word groups were selected from a previous study (Marriage et al., 2011) and were designed to be equally discriminable for normal-hearing listeners. Figure 4(a) shows the ability of listeners in Experiment 1 to discriminate words in each of the four groups, with performance averaged across all spatial locations. Performance across the four word groups was statistically indistinguishable, *F*(3,27) = 0.67, *p* = .58. Figure 4(b) plots the relative localization performance for the same four word groups, again averaged across all spatial locations. In contrast to word identification performance, relative localization performance depended on the word group, *F*(3,27) = 11.1, *p* < .001. Performance in the consonant final (Cf) group was significantly higher than in the other three groups (Figure 4(b)).

**Figure 5.**
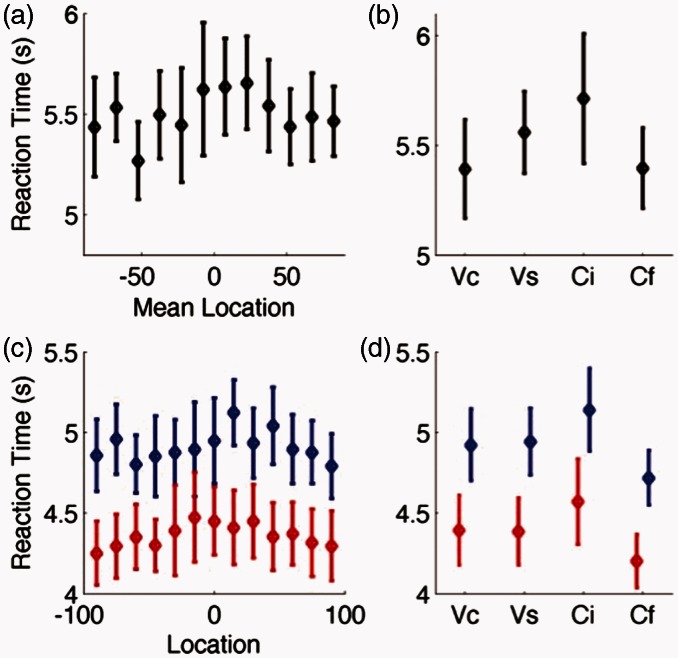
Reaction time data for Experiment 1. (a, b) Reaction times (in seconds) for relative localization responses plotted according to their mean location (a) or word group (b). (c, d) Reaction times for word identification judgments by spatial location (c) or word group (d). Reaction times for the first speech token are shown in red and for the second word token in blue.

We considered participants’ reaction times in addition to their accuracy because it was expected that various spatial locations would have different impacts on difficulty and those distinctions that were harder to detect would be associated with a longer reaction time. Figure 5 displays reaction times for both the relative spatial discrimination aspects of the task (a and c) and the speech identification elements of the task (b and d). While participants were free to respond in whatever order they were most comfortable, all 10 of our listeners chose to identify the two target words before responding to the spatial discrimination element of the task. While there is a trend for reaction times to be longer near the midline in both tasks, there were no significant differences in reaction time in either task whether considered by spatial location (repeated measures ANOVA; spatial discrimination, *F*(11,99) = 1.31, *p* = .31; word identification, *F*(12,99) = 0.73, *p* = .71) or word group (repeated measures ANOVA; spatial discrimination, *F*(3,27) =1.69, *p* = .19; word identification, *F*(12,99) = 2.67, *p* = .07).

**Figure 6.**
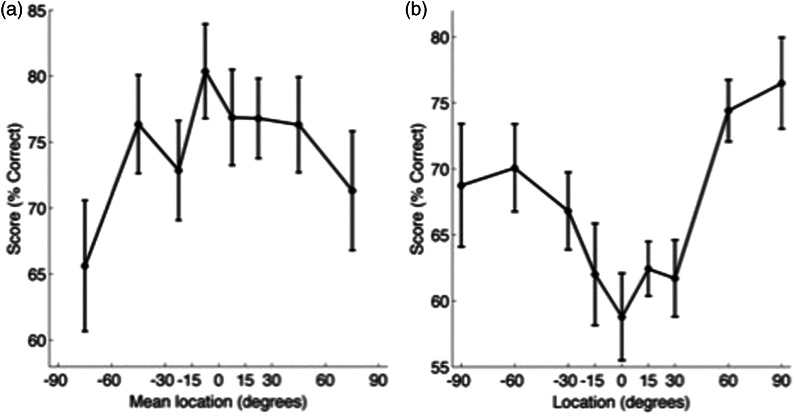
Experiment 2, reduced speaker array. (a) Relative localization performance (mean ± *SE*, % correct) by mean speaker location. (b) Word identification performance by location.

**Figure 7.**
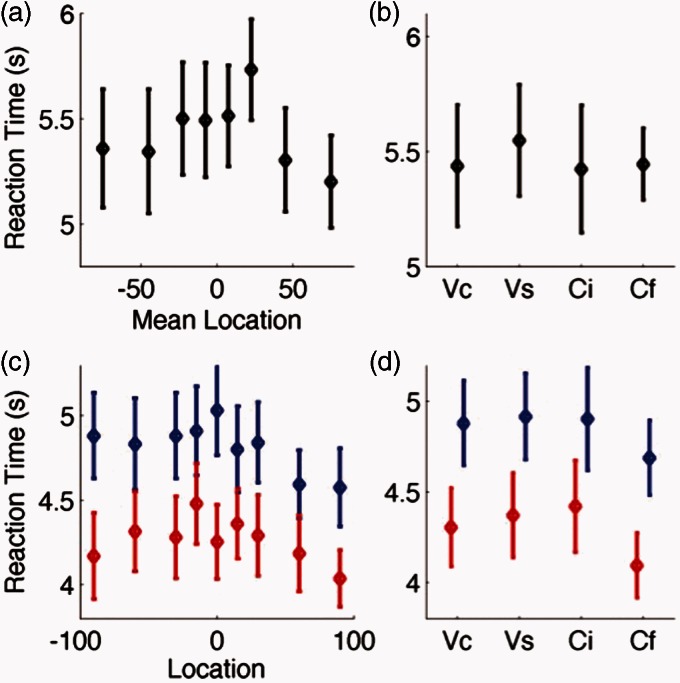
Reaction time data for Experiment 2. (a, b) Relative localization responses plotted according to their mean location (a) or word group (b). (c, d) Reaction times for word identification judgments by spatial location (c) or word group (d). Reaction times for the first speech token are shown in red and for the second word token in blue.

Finally, in Experiment 2, we compared performance using a reduced set of speaker locations, equivalent to those found in the Crescent of Sound. In Experiment 1, speakers were separated by 15°. In Experiment 2, the front five speakers (−30° : +30°) were separated by 15°, and the more lateral four speakers (−90° : −30° and +30° : 90°) were separated by 30°. The effects of spatial location on word identification from Experiment 1 were reproduced (Figure 6): Speech discrimination was better at more peripheral locations (repeated measures ANOVA for the effect of speaker location on word identification, *F*(8,72) = 6.81, *p* < .001 (Figure 6B)). Relative spatial discrimination did not differ significantly according to speaker location, *F*(7,63) = 1.86, *p* = .09 (Figure 6A). As in Experiment 1, all word groups were equally discriminable, *F*(3,27) = 0.16, *p* = .93, but the Cf group produced better spatial discrimination performance, *F*(3,27) = 29.4, *p* < .001. Similar trends in reaction time were evident in Experiment 2 as Experiment 1 (Figure 7), but as in the previous experiment, there were no significant differences in reaction time across either spatial locations or word groups.

Finally, to determine the test–retest reliability of the simultaneous spatial discrimination and speech identification test, an intraclass correlation analysis was performed, using a two-way random effects model, type consistency. To perform this analysis, proportion correct speech identification or spatial discrimination scores were calculated from the first and second half of the trials, having first collapsed across spatial location. Analysis was performed separately for Experiment 1 and Experiment 2. For the speech identification scores, the Cronbach’s alpha scores were .97 and .69 for Experiment 1 and Experiment 2, respectively. For the spatial discrimination measures, the Cronbach’s alpha score was .87 and .73 for Experiments 1 and 2, respectively. The reliability scores for Experiment 2 were lower than Experiment 1 when the first and second halves of the samples were used; however, these values are acceptable for the development of an assessment where .70 is considered acceptable (Nunnally, 1978). The appropriate number of presentations of stimuli could be somewhere between the number of presentations used in half of Experiment 1 (96 items) and Experiment 2 (65 items). In summary, these tests offer satisfactory test–retest reliability, and, given that these measures were calculated from half of the dataset, it suggests the potential to shorten and potentially half the number of test items and in turn the duration of the task.

## Discussion

The goal of this study was to establish a procedurally simple task that allowed simultaneous assessment of speech recognition and spatial abilities in one single efficient task. To achieve this aim, we designed a task in which listeners heard two sequentially presented words, embedded in a multisource noise background, and had to perform a single-interval two-alternative forced choice localization judgment and identify both words from a closed set of four alternatives. Everyday listening environments are dynamic, with a wide range of spatial sources contributing to the soundscape. This test requires that an individual can understand speech from unpredictable locations and judge the relative location of the two speech sounds. As such it is therefore a good indicator of how well they can use their spatial hearing.

The task was developed by carefully incorporating selected stimuli from the CAPT speech identification task into the context of a recently developed relative localization task (Wood & Bizley, 2015). In addition to requiring listeners to determine the relative location of the two sequentially presented words, listeners were also required to identify the words. One of the underlying principles for the spatial discrimination task was to assess spatial listening using a two-alternative forced choice procedure with speakers at a fixed angular separation because it enabled rapid assessment of spatial discrimination throughout azimuthal space. Unlike the majority of spatial discrimination tasks where listeners are required to localize or discriminate artificial stimuli such as pure tones or broadband noise, this task used speech stimuli as the target items, and these were presented in a background of multisource babble. The measures of spatial discrimination provided therefore give a more ecologically valid estimate of localization ability, which may relate more closely to their ability to use spatial cues in everyday. The use of speech stimuli should have the impact of making the task more appropriate for children who would find speech tokens a more interesting and meaningful stimulus than, for example, noise bursts. The use of a two-alternative forced choice design is also ideally suited to groups of listeners, such as binaural CI users, who may have relatively poor spatial acuity, because it makes the response approach easier. Nevertheless, the pattern of results in Experiment 1 for spatial discrimination observed using speech stimuli is very similar to that observed with broadband noise in a previous study conducted by Wood and Bizley (2015). In the Wood and Bizley study, spatial discrimination ability was assessed with broadband or spectrally restricted noise. While performance was superior in and around the midline in all cases, in the spectrally restricted conditions, there was a much more marked decrease in listening ability at more eccentric speaker locations. The similarity between broadband performance in Wood and Bizley and the performance with speech in Experiment 1 presumably reflects the availability of both binaural level and timing cues present in a spectrally rich stimulus such as speech. The improvement in performance close to the midline reflects the superior availability of binaural localization cues, whereby a 15° shift in location elicits larger changes in binaural cue values when the shift occurs close to the midline than when the same shift occurs in the periphery (Mills, 1958; Shaw & Vaillancourt, 1985; Wood & Bizley, 2015).

Experiment 1 used small differences in speaker separation to assess spatial discrimination because normal-hearing adult listeners were performing the task and demonstrated that word identification was superior in the periphery, and spatial discrimination was best around the midline. In Experiment 2, a wider speaker spacing was used at more peripheral locations, to match that currently clinically available in the Crescent of Sound. In this experiment, listeners showed superior word identification performance at peripheral compared to central locations. However, the spatial listening results were not significantly different throughout space, presumably because the speaker spacing was too coarse to detect differences in sensitivity as a function of azimuthal angle. It is recognized in going forward that hearing-impaired and also younger populations are likely to have worse spatial discrimination abilities and that larger spatial step sizes may be required. Likewise, if listeners indicate very high levels of performance, it may be necessary to decrease the speaker separation to ensure that all ranges of ability can be assessed, thus ensuring that the test is applicable to a variety of listeners including pediatric CI users, or elderly hearing aid users.

Listeners’ ability to correctly identify speech in multitalker babble varied systematically with azimuthal location. In contrast to the spatial discrimination task, where performance was superior at or around the midline, in the speech identification task, listeners were best able to accurately identify the words when they arose from the speakers located away from the midline. Improvements in speech reception thresholds away from the midline likely result from both monaural and binaural factors. An off-midline sound will, due to the head shadow, bring about a monaural advantage by enhancing the SNR at the near ear (the *better ear* effect). Binaural cues (interaural level differences and interaural time differences) may also enhance target audibility through binaural interaction (Colburn, 1977; Durlach, 1972; Edmonds & Culling, 2006; Hawley, Litovsky, & Culling, 2004; Levitt & Rabiner, 1967).

Listeners discriminated four sets of words, each selected to probe a different phonetic contrast. While listeners were equally able to identify words across all four groups, they were significantly better at spatially discriminating words in which the phonetic contrast was the final consonant. This could relate to the fact that for the final consonant stimulus the listener knew that the acoustic cue difference was at the end of the word giving them time to concentrate on the relative localization decision before considering the word identification. For voiceless consonant word initial contrasts the first phoneme often has reduced acoustic parameters compared to word final position, such as F1 cutback (Jiang, Chen, & Alwan, 2006) making the identification judgment harder, therefore reducing the time available for making the location judgments.

Before embarking on the main test, listeners performed a threshold test that allowed us to match difficulty across listeners such that listeners’ performance fell above chance and below ceiling. Previous investigations that have measured spatial listening abilities in hearing-impaired children have often struggled to draw meaningful conclusions due to floor and ceiling effects (Lovett, Vickers, & Summerfield, 2015). By including this threshold stage, we were therefore able to ensure that listeners were operating at the same point on the psychometric function and therefore allowed us to measure differences in performance across auditory space in both speech identification and sound discrimination tasks. We set this threshold based on the speech discrimination ability, and this allowed us to observe performance within the range that we determined would be most meaningful (43% to 82% correct, see Methods section). Given the variability likely to be observed among clinical populations, it will be advantageous to be able to adjust test difficulty for each individual so that we can maintain the sensitivity of the test to variations in performance across space and across different listening conditions. While this approach was successful in normal-hearing listeners, pilot experiments will have to be performed before we can conclude that this approach allows us to measure performance in hearing-impaired listeners. Spatial listening abilities in bilateral CI users are worse than those of normal-hearing listeners with typical error magnitudes of 10° in the midline and 28° in the periphery (Mantokoudis et al., 2011). It seems likely that the larger speaker separations tested in Experiment 2 might be better suited to test patient groups than normal-hearing listeners. Should pilot experiments reveal that coarser (or finer) speaker separations are necessary to ensure that hearing-impaired listeners are above chance (and below ceiling), it will be straightforward to vary the speaker separations. The overall duration of testing for each participant was ∼20 min for the threshold test and 35 or 25 min for Test 1 and Test 2, respectively. As Test 2 was designed to mimic a clinical setting, this means that testing can easily be completed within an hour—however, given that the test–retest reliability measures were calculated based on only half of the available data, there may be potential to shorten the test further and decrease its duration it is estimated (estimated that 15 min would suffice). Further piloting to measure reliability with shorter test batteries, including performing testing at two different time points, is desirable before this test can be used clinically.

The use of speech stimuli and a simple, intuitive touch screen makes this task both simple to perform and engaging for listeners. The results demonstrate that this single test provides a useful measure of both speech identification in noise and spatial discrimination in a complex, realistic listening condition. In its current form, the test would potentially be suitable for a wide range of patient groups and with relatively simple adaptation could be suitable for younger children. Additional experiments with such patient groups are required to assess its suitability and fine-tune the experimental parameters and speaker arrangements.
